# Transarterial Radioembolization (TARE) in Patients with Hepatocellular Carcinoma: A Comparison of Palliative with Bridging-to-Transplant Concepts

**DOI:** 10.3390/cancers16010235

**Published:** 2024-01-04

**Authors:** Jacqueline Schönherr, Philipp Seifert, Falk Gühne, Thomas Winkens, Falk Rauchfuß, Utz Settmacher, Martin Freesmeyer, Robert Drescher

**Affiliations:** 1 Clinic of Nuclear Medicine, Jena University Hospital, 07747 Jena, Germany; jacqueline.schoenherr@med.uni-jena.de (J.S.); philipp.seifert@med.uni-jena.de (P.S.); falk.guehne@med.uni-jena.de (F.G.); thomas.winkens@med.uni-jena.de (T.W.); robert.drescher@med.uni-jena.de (R.D.); 2 Center of Transplant Surgery, Department of General, Visceral and Vascular Surgery, Jena University Hospital, 07747 Jena, Germany; falk.rauchfuss@med.uni-jena.de (F.R.); utz.settmacher@med.uni-jena.de (U.S.)

**Keywords:** liver carcinoma, hepatocellular carcinoma, radioembolization, liver transplantation, bridging therapies

## Abstract

**Simple Summary:**

Liver transplantation (LT) is the best potentially curative treatment for unresectable, early-stage hepatocellular carcinoma (HCC) in cirrhotic patients. Bridging-to-transplant therapies, including transarterial radioembolization (TARE), are performed to delay tumor progression until the LT can be performed but also offer potential benefits regarding post-transplant progression-free survival. This study confirms the good results of bridging-to-transplant TARE, of LT as a curative treatment of HCC, and also shows the importance of TARE in the palliative, multimodal treatment of patients with HCC.

**Abstract:**

We investigated transarterial radioembolization (TARE) as a palliative measure and bridging-to-transplant therapy in hepatocellular carcinoma (HCC) patients. A total of 167 patients (50 bridging, 117 palliative) with 245 TARE procedures were assessed. Fourteen patients underwent subsequent liver transplantation (LT). Patients undergoing LT exhibited significantly prolonged progression-free survival (PFS) compared to those with bridging-without-transplant (*p* = 0.033). No significant differences were observed between patients with bridging-without-transplant and palliative cases (*p* = 0.116). Median overall survival (OS) post-TARE was 16.6 months, with estimated OS rates at 6/12 months of 82.0%/60.5%, respectively. Patients who underwent LT demonstrated statistically significantly longer OS compared to those with bridging-without-transplant (*p* = 0.001). No marked outcome distinctions were found between bridging-without-transplant and palliative groups. The findings underscored the superiority of LT over alternative treatments. TARE served as an important component in non-LT scenarios, allowing for subsequent therapeutic options. The study reflected the highly variable and complex situations of patients with HCC, emphasizing the need for further investigations to define an optimal multimodal approach.

## 1. Introduction

Hepatocellular carcinoma (HCC) represents the most prevalent primary malignant liver tumor globally. It ranks sixth in worldwide tumor diagnosis frequency and third in global cancer-related fatalities for both genders [1]. Liver transplantation (LT) remains the only treatment option capable of eliminating malignancy while addressing underlying liver cirrhosis. In Europe, the waiting time for an organ from a deceased donor varies from 6 to more than 12 months, depending on urgency and donor organ availability [2]. During this period, a relevant proportion of patients withdraw due to tumor progression or other diseases [3]. For patients awaiting transplantation, it is recommended to bridge with locoregional therapies [4,5]. While delaying tumor progression, the response to locoregional bridging therapy can serve as a vital tumor-biological selection criterion over time. It effectively sifts out biologically aggressive tumors while patients await a graft, resulting in a more precise selection of transplant beneficiaries [6,7,8]. Notably, patients are not left untreated, even if they are not undergoing LT.

Transarterial radioembolization (TARE), also known as selective internal radiation therapy (SIRT), is performed to treat primary malignancies and liver metastases. The therapy is based on the predominant arterial vascularization of tumors compared to non-tumoral liver tissue. When microspheres containing beta-emitting nuclides are administered via a microcatheter into the artery feeding the tumor-containing liver tissue, the resulting tumor doses are higher than those delivered to non-tumor liver tissue [9,10]. In addition to palliative treatments, TARE can also be performed as a bridging therapy to delay hepatic tumor progression and to ensure that patients survive the waiting time for a liver transplant without developing contraindications for LT [11,12].

Three types of microspheres are available which are approved for the locoregional treatment of HCC:

- ^90^Y-containing resin microspheres (SirSpheres^®^, Sirtex Medical, Woburn, MA, USA);
- ^90^Y-containing glass microspheres (TheraSphere^®^, Boston Scientific, Marlborough, MA, USA);
- ^166^Ho-containing poly-l-lactic acid (PLLA) microspheres (QuiremSpheres^®^, Terumo, Leuven, Belgium).

The primary objective of this study was to systematically evaluate the outcome of patients who underwent bridging-to-transplant TARE in our institution. Furthermore, a comparison was made between patients who underwent bridging-to-transplant TARE but not LT with patients treated in a palliative setting.

## 2. Materials and Methods

The study received approval from our institutional ethics committee (registration number: 2020-1908). All participating patients provided written consent for the anonymous use of their data for research purposes when they sought treatment at our hospital.

### 2.1. Inclusion Criteria and Patient Characteristics

All patients with HCC who consecutively underwent TARE treatments from the introduction of this method at our hospital in 2011 to 2020 were included. The clinical indication for TARE was determined by a multidisciplinary tumor board (MDT), which also assessed a patient’s eligibility for liver transplantation and the need for bridging-to-transplant treatment.

Demographic and clinical data, including tumor diagnosis, concurrent diseases, treatments before and after TARE procedures, and follow-up information, including reports and imaging, to assess progression-free survival (PFS) and overall survival (OS) were analyzed. Disease progression on imaging was defined using the modified Response Evaluation Criteria in Solid Tumors (mRECIST) [13]. Follow-up was conducted until 12 months after the inclusion of the last patient in the study, or until their death, for all previously enrolled patients. Comparisons were made with the data of patients who underwent TARE in a palliative setting.

### 2.2. TARE Procedures

All TARE procedures were performed according to the manufacturer’s recommendations and comprised an angiography of the hepatic vasculature, planar scintigraphy of the thorax and abdomen, and a single-photon emission computed tomography (SPECT) combined with computed tomography (CT) of the abdomen [14,15,16]. ^99m^Tc-labeled human serum albumin (HSA) B20 microspheres (ROTOP, Dresden, Germany) were used for TARE simulations to determine activity distribution in and outside the liver and to calculate the lung shunt fraction. TARE treatment procedures were performed 1–2 weeks after planning. Activity calculation for ^90^Y-resin microspheres was conducted with the multi-compartment/modified body surface area (BSA) method [16]. For ^90^Y-glass and ^166^Ho-PLLA microspheres, calculations were carried out with the respective single-compartment formulae [14,15].

### 2.3. Follow-Up

Post-procedure, patients stayed in a nuclear medicine ward for 24 h (TARE planning) or 48 h (TARE treatment) and underwent scintigraphy and SPECT/CT to confirm the distribution of the microspheres in the body. The first imaging follow-up was scheduled three months after completion of the TARE procedure(s) and provided a basis for further treatment planning and surveillance.

### 2.4. Outcome Evaluation and Statistics

Overall survival (OS) was defined as the interval from the first TARE procedure to the time of death (or to the end of follow-up for patients still alive). Progression-free survival (PFS) was defined as the interval from the first TARE procedure to disease progression on imaging, death, or at the end of follow-up.

Characteristics between patient groups were compared with *t*-tests for continuous variables and chi-square tests for noncontinuous variables. Survival outcomes were analyzed with Kaplan–Meier methods. Statistical analyses were conducted using SPSS Statistics (IBM, Armonk, NY, USA) and Stata/IC (Stata Corporation, College Station, TX, USA). A *p*-value of <0.05 was considered significant.

## 3. Results

### 3.1. Clinical Characteristics and Indications for TARE Treatment

**Figure 1:**
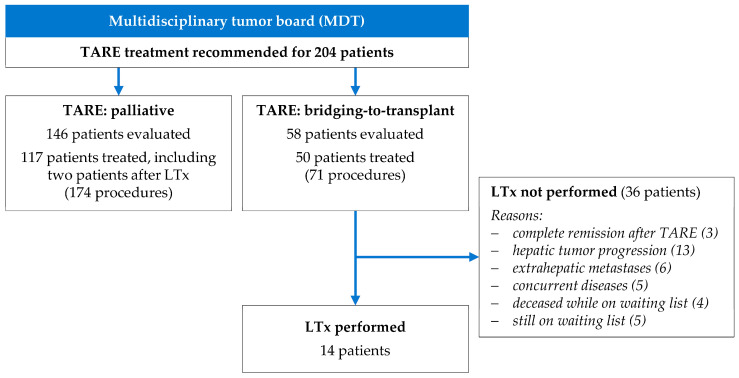
Clinical indication of TARE as palliative treatment and as bridging-to-transplant.

In the study period, the MDT recommended TARE treatments for 204 patients with HCC (Figure 1). A total of 37 patients (18.1%) who were evaluated did not undergo TARE treatment due to insufficient activity accumulations in tumor lesions (*n* = 13), extrahepatic activity accumulation (*n* = 6), high lung shunt (*n* = 5), deterioration of liver function in the interval (*n* = 7), or unfavorable vascular anatomy for TARE (*n* = 6).

A total of 167 patients who underwent 245 TARE procedures were included in the study (Table 1). In 50 patients (29.9%), TARE procedures were performed as bridging treatment to LT. The 117 patients (70.1%) who were considered ineligible for LT by the MDT underwent palliative TARE procedures.

Table 1 displays the clinical characteristics of the patient groups before TARE. Patients in the bridging-to-transplant group were younger than those in the palliative group with median ages of 62.6 and 72.0 years, respectively. There were no significant differences in other parameters, HCC stage, or Child–Pugh scores. The treatment sequences for HCC before TARE were diverse: TARE was the first-line treatment for 46.0% of patients in the bridging-to-transplant group and 51.3% of patients in the palliative group, respectively. The most common treatments before TARE were surgery and TACE.

### 3.2. TARE Interventional Procedures

A total of 245 TARE procedures were conducted within 209 treatment cycles (Table 2). Of these, 36 TARE cycles included the whole liver (72 procedures, bilobar sequential approach with separate TARE procedures of both liver lobes, interval of 5–6 weeks). In 173 TARE cycles, only one liver lobe was treated. A total of 113 procedures (64.5%) were performed with ^90^Y-glass, 51 procedures (34.3%) with ^90^Y-resin, and 3 procedures (1.2%) with ^166^Ho-PLLA microspheres.

The tumor burden within the target volume was statistically significantly lower in the bridging-to-transplant group compared to the palliative group with medians of 5.6% and 8.8%, respectively. However, no significant differences between the groups were observed concerning other parameters.

### 3.3. Liver Transplantation and TARE

Among the 50 patients who underwent bridging-to-transplant TARE, 14 patients (28%) proceeded to liver transplantation (Figure 1). The median interval between TARE and liver transplantation was 5.6 months (range 0.5 to 25.5 months). In three patients, TARE was the sole treatment before LT. The median time on the transplant waiting list was 7.3 months (range 0.9 to 21.6 months). Of these transplantations, 12 were deceased-donor and 2 were living-donor procedures, all performed without procedural complications.

Twelve months after liver transplantation, 13 out of 14 patients were still alive. By the end of the follow-up period, 11 out of 14 patients were still living. A 61-year-old man underwent liver transplantation 2.6 months after a right lobar TARE but deceased 2.5 months later due to an acute inferior vena cava thrombosis. A 71-year-old man suffered from fatal sepsis with hepatic abscesses 14.4 months after liver transplantation. A 62-year-old man died 31.3 months after LT by extrahepatic tumor progression.

A total of 36 of the 50 patients (72%) who underwent bridging-to-transplant TARE did not undergo LT during the duration of the study (Figure 1). In 19 patients, a local and/or metastatic tumor progression was detected, and five patients had concurrent diseases preventing transplantation (such as newly diagnosed gastric cancer, progressive coronary artery disease, recurrent variceal bleeding, and renal disease/sepsis). In three patients (aged 56–67 years; two with bilobar sequential and one with right lobe treatment; tumor load 3–8%; ^90^Y glass microspheres), follow-up CT three months after TARE showed complete HCC remission.

Two patients underwent TARE after living-donor liver transplantation. Recurrent HCC was detected 25.5 and 40.9 months after transplantation, respectively. In the first patient, a 71-year-old man, HCC recurrence was treated with radiofrequency ablation followed by sorafenib. After the detection of further HCC progression, TARE of the transplanted liver with ^90^Y glass microspheres was performed and a progression-free survival (PFS) of 8.9 months was achieved, after which sorafenib was reintroduced. The second patient, a 66-year-old man, underwent transarterial chemoembolization (TACE) for a solitary recurrent lesion, but multiple additional HCC lesions developed 11 months later. A subsequent right lobar TARE with ^90^Y resin microspheres achieved a PFS of 7.7 months. No further treatment was initiated.

### 3.4. Progression-Free and Overall Survival

The median follow-up time for patients in this study was 14.5 months (range 0.9–112.6 months) after TARE and 26.8 months (range 2.5–94.8 months) after LT. A total of 39.5% of patients underwent additional HCC treatments after TARE, mostly with locoregional methods including TACE and percutaneous radiation (Table 3); 20.4% of patients received systemic therapy.

**Figure 2:**
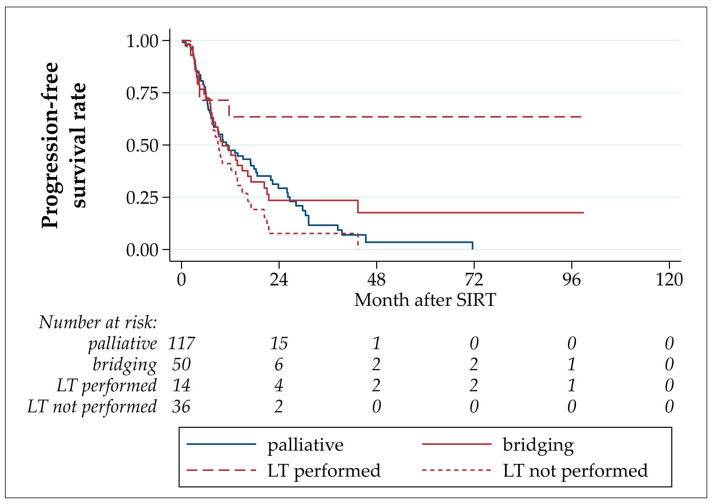
Progression-free survival after TARE.

In the overall cohort, the median PFS after TARE was 11.0 months, with estimated PFS rates after 6 and 12 months of 73.4% and 47.1%, respectively (Table 3 and Figure 2). The PFS of patients who underwent LT was statistically significantly longer than those of patients who underwent bridging TARE but not LT (*p* = 0.033; hazard ratio LT not performed/LT performed: 2.81 (CI 1.08–7.29)). In four patients, an intrahepatic progression was detected before LT (three in TARE-treated segments, one in untreated segments). No intrahepatic tumor recurrence after LT was observed. Three patients developed extrahepatic metastases after LT: in the abdominal wall musculature (92 months after TARE, 79.1 months after LT); adrenal, lung, and lymph node metastases (17.3 months after TARE, 12.3 months after LT); and peritoneal and lung metastases (11.7 months after TARE, 5.9 months after LT).

No statistically significant differences were detected by comparing patients who underwent bridging-without-transplant LT to palliative patients (*p* = 0.116, hazard ratio LT not performed/palliative: 1.40 (CI 0.92–2.13)), and palliative with all bridging TARE patients (*p* = 0.932, hazard ratio palliative/bridging: 1.02 (CI 0.67–1.54)).

**Figure 3:**
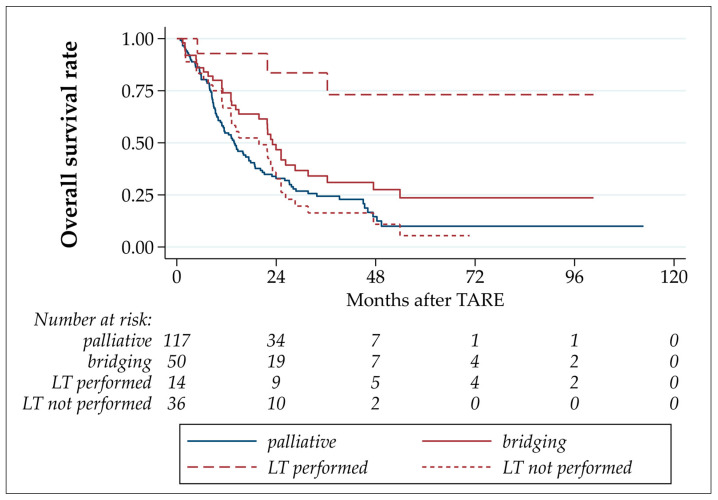
Overall survival after TARE.

The median OS after TARE in this study was 16.6 months, with estimated OS rates after 6 and 12 months of 82.0% and 60.5%, respectively (Table 3 and Figure 3). The OS of patients who underwent LT was statistically significantly longer than those of patients who underwent bridging-without-transplant (*p* = 0.001; hazard ratio LT not performed/LT performed: 7.68 (CI 2.30–25.61)). No statistically significant differences were detected by comparing patients with bridging-without-transplant to palliative patients (*p* = 0.9666, hazard ratio LT not performed/palliative: 0.99 (CI 0.68–1.45)). The comparison of palliative with all bridging TARE patients yielded a statistically significant difference caused by the well-performing LT patients in the bridging group (*p* = 0.029, hazard ratio palliative/bridging: 1.55 (CI 1.05–2.30)).

## 4. Discussion

Over the years, transarterial radioembolization (TARE) has evolved into an established component of the treatment regimen for patients with hepatocellular carcinoma (HCC). Positioned as a locoregional therapeutic method, it occupies an intermediate position between surgery as a curative approach, local therapy options (such as transarterial chemoembolization (TACE) and radiofrequency ablation (RFA)), and systemic therapy. TARE as locoregional therapy is indicated when large and/or multiple HCC lesions are present, which can no longer be individually addressed, and when there is no prognostically relevant extrahepatic metastatic spread.

The decision regarding the precise therapy for an individual patient is made in multi-disciplinary tumor boards (MDT). Medical experts from various fields collaboratively establish comprehensive management plans based on consensus, current guidelines, and scientific knowledge. An important factor in the decision for a treatment option is the liver function state of the patient. For patients with hepatocellular carcinoma (HCC), either curative or palliative therapy may be indicated, or a treatment inherently defined as palliative is applied to facilitate subsequent curative interventions. In this context, a bridging therapy, such as transarterial radioembolization (TARE) before liver transplantation (LT), is applied. The effectiveness of locoregional, neoadjuvant therapies for this purpose has been demonstrated by several studies [5,6,11,17,18,19,20,21,22,23,24,25]. Studies directly comparing TARE with TACE found a higher disease control rate, significantly better overall and intrahepatic PFS, and better survival outcomes in the patients treated with TARE even with advanced disease [19,20]. An important advantage of TARE appears to be the possible induction of contralateral hepatic hypertrophy and the feasibility of patients with portal vein thrombosis (PVT) [18,19]. A recent review concluded that TARE is a feasible treatment option to save patients for LT if it meets established indication criteria [12]. In the three studies reporting on the effect of bridging to transplant for HCC included in this review, favorable outcomes were observed [26,27,28].

### 4.1. Clinical Outcome

The estimated survival rate in our whole patient cohort was 82.0% and 60.5% at 6 and 12 months after TARE, respectively, which is similar to other studies evaluating patients with unresectable HCC, also regarding the proportion of patients who underwent LT after bridging TARE [11,23,24]. Despite our hospital being a specialized liver transplantation center, only approx. 1/3 (29.9%) of all TARE are performed for bridging, and only a proportion of these patients (28%) actually underwent transplantation. In most cases, this is explained by a tumor progression (intrahepatic and/or metastatic) or diagnosis of concurrent diseases, for some patients even preventing their inclusion on the transplant waiting list. Regarding confounding factors, worse overall and progression-free survival was related to a higher tumor burden and Child–Pugh score. The absence of liver cirrhosis, presence of tumor response, and curative treatment following TARE were identified as predictors of both OS and PFS, while tumor size independently predicted tumor response [23]. An earlier study from our own hospital which partially overlaps the patient cohort in this study compared patients undergoing LT with and without bridging treatment (most commonly TACE and RFA; TARE in only 8% of patients) [17]. The 5- and 10-year survival rates for patients with bridging were 67% and 47% and without bridging were 56% and 46%, respectively. Tumor-related 10-year survival showed a statistically significant difference between both groups (81% versus 59%).

TARE with ^166^Ho-PLLA microspheres was introduced in our hospital in 2019, and only three patients were included in this evaluation [29]. Holmium-166 has a shorter half-life than yttrium-90 (26.8 and 64.1 h, respectively), resulting in higher tissue dose rates. If this is of advantage for the treatment of HCC, or of more aggressive HCC subtypes, is still unclear. Also, studies regarding the time of best response comparing ^166^Ho- with ^90^Y-TARE are not available yet.

### 4.2. Comparison of Bridging to Palliative Treatments

Our patients in the bridging-to-transplant group were younger (median age 62.6 and 72.0 years, respectively) and had lower tumor burden in the treated liver (median 5.6% and 8.8%, respectively) than those in the palliative group (Table 1 and Table 2). Therefore, a better outcome not only of the patients who underwent LT but also of patients who underwent bridging-without-transplant was expected. However, the lower tumor burden in the bridging TARE group did not translate to an unequivocally better clinical outcome for patients who did not undergo LT. After bridging TARE without LT, the median PFS was shorter (9.2 and 11.4 months, respectively) and the median OS was longer (19.8 and 13.9 months, respectively), with both differences being not statistically significant. In our patients, age and tumor burden in the liver at the time of TARE were not clear prognostic factors.

In the bridging-to-transplant group, there were fewer patients with CPS stage B than in the palliative group (6.0% and 11.1%, respectively; not statistically significant); no patients with CPS stage B underwent LT.

Locoregional intra-arterial therapies are increasingly performed in sequence or combination with other locoregional or systemic treatment options and should be applied according to disease state, progression, liver function, and concurrent diseases. A multimodal and multidisciplinary approach yields the best oncologic results, and TARE should be seen as a complement, not a competitor, to other therapies [22,30]. Improved PSF has been shown for a combination TACE/sorafenib compared with monotherapy [31]. The same may be valid for combinations with TARE based on a proposed synergistic mechanism between radiation and the effects of immune checkpoint inhibitors [32]. A phase 2 study assessing the efficacy of local tumor control in HCC patients who receive ^90^Y-glass TARE followed by durvalumab and tremelimumab is enrolling patients (ROWAN trial, NCT05063565). Since any previous treatment may impact the functional reserve of the liver, the functional parameters should be evaluated with the greatest care, using established functional scores [33]. Additional checks, including liver maximum capacity test (LiMAx) and hepatobiliary scintigraphy, may be helpful tools for assessment [34,35].

A relatively rare setting is to perform a TARE in patients with HCC recurrence after LT, but it has been described as a feasible option [36]. In our patient cohort, two patients were successfully treated with ^90^Y-TARE after LT without complications. The liver function remained stable.

### 4.3. Outcome of Patients Undergoing Liver Transplantation

In the patients with LT in our study, we observed an estimated 12-month survival rate of 92.9%. The main limitation to survival was the occurrence of extrahepatic metastases: of the five patients who underwent bridging TARE followed by LT and in whom tumor progression was detected, three patients developed extrahepatic metastatic spread after LT. Two of the patients with metastases also had intrahepatic progression after TARE but before LT (one in liver segments treated by TARE, one in the untreated liver). Since in one of these patients the metastatic spread occurred more than five years after TARE and LT, a correlation cannot be postulated. In the literature, intrahepatic HCC recurrences are described for 10–20% of patients after LT, also depending on the success of the bridging therapy applied before LT. Despite being not evident in our cohort without HCC recurrence during the follow-up period, bridging treatments probably lower the risk of HCC recurrence after liver transplantation [18,19]. In a study including 207 patients who underwent LT after ^90^Y-TARE, long-term OS rates were similar to LT for non-malignant liver disease. It was hypothesized that the low rate of HCC recurrence in liver transplants is also attributable to TARE effects [19].

### 4.4. Future Perspectives

Image-guided locoregional therapies (LRTs) for primary liver tumors must be seen in the context of the heterogeneous nature of HCC with various subtypes and tumor microenvironments [37]. Therapies such as TARE are not targeted on a molecular level but are vascularly targeted by injecting radioactivity directly into the tumor or, at least, into the artery supplying the tumor-containing liver tissue. In this manner, LRTs may maintain their role in the treatment sequence, combined with molecular-targeted substances that have significantly increased the life expectancy of patients with advanced HCC [22]. The currently recommended first-line therapy for advanced-stage HCC is a combination of immune checkpoint and tyrosine kinase inhibitors (e.g., atezolizumab/bevacizumab), but the most effective second-line options may be combinations of systemic and locoregional therapies [38,39].

Due to the application of radioactivity at the capillary level and the damage to endothelial cells, TARE may be particularly effective in preventing the invasion of healthy tissue by tumor neovasculature [21,40]. This would also account for a low extrahepatic recurrence rate after TARE, as the number of circulating HCC cells may decrease. Further studies involving the detection of tumor cells and DNA in the blood would be necessary to address this question. Under investigation are methods to sensitize radiotherapy by depressing PD-L1 expression and reversing tumor hypoxia, since PD-L1 may be upregulated secondary to radiation, thus limiting response to treatment. The antineoplastic agent lonidamine (LND) is brought into the cells using nanoparticles [41,42]. Prospective individualized TARE planning and treatment with multi-compartment, voxel-based dosimetry models may improve clinical outcomes. It allows a prediction of tissue doses when performing TARE and thus an adjustment of the dose to each individual patient setting. Dose–response relationships for the treatment of HCC with ^90^Y-glass microspheres and the treatment of CRC metastases with ^166^Ho-PLLA microspheres have already been established [43,44]. A ^166^Ho-TARE dose-finding study for early-stage HCC is ongoing [45]. For all three available types of microsphere, recommendations exist for conducting the treatments, which also include information on dosage planning, the target dosage on the tumor, and the preservation of non-tumor-affected liver tissue [46,47,48]. However, these are mostly based on retrospective data. Future studies should aim to define dose thresholds in different clinical situations.

A very important step beyond bridging-to-transplant is the inclusion of TARE as a treatment option for early- and intermediate-stage HCC [39]. In these patients, TARE is now recommended if resection, ablation, or LT are not successful or not feasible. Based on the results of the LEGACY study, TARE is also recommended for solitary HCC with a diameter of up to 8 cm [49]. These changes, not yet included in AASLD and EASL guidelines [4,50], represent a paradigm change away from the application of TARE as a last attempt at treatment, often indicated by an MDT when all other methods were no longer available. It is obvious that during TARE planning, the focus should now be laid on the preservation of liver function so that TARE does not prevent further treatments, in particular, the growing systemic options. In patients in which it is not possible to reach a perfect tumor-to-liver activity ratio, limiting the dose to the healthy liver tissue instead of maximizing the tumor dose should be considered, therefore preserving the possibility of further TARE treatments over the clinical course of the patient and preserving sufficient liver function reserves [33,51]. New HCC lesions may occur in previously tumor-free liver segments, and further liver-function-impairing treatments may be necessary.

### 4.5. Limitations of the Study

The main limitation of the study is that it is a retrospective observation of the highly variable oncologic treatment sequences of patients with HCC in a single center, not a controlled study. The outcome evaluation focuses on TARE procedures and therefore on the nuclear medicine/interventional radiology view. Since the majority of patients received other treatments before and/or after TARE, treatment effects cannot be attributed to a single method. In view of the high variability of the treatment sequences, survival calculations were carried out in relation to the date of the TARE treatments. A comparison with other treatment options (e.g., TACE, percutaneous radiation) was not performed.

## 5. Conclusions

In the presented study, we evaluated the outcome of TARE procedures performed in our hospital in the context of LT. The indication of a TARE procedure as a bridging or a palliative treatment was set in the MDT. PFS and OS analyses and comparisons between the groups bridging-with-transplant, bridging-without-transplant, and palliative confirmed the advantage of LT in comparison to other treatment sequences. In patients who did not undergo LT, TARE was an important part of the multimodal HCC treatment sequence. No statistically significant outcome differences were detected between bridging-without-transplant and palliative groups. The future of HCC treatment probably lies in the combination of locoregional and systemic therapies, whose exact application still needs to be clarified. Current and future studies for TARE should address the optimization of dosimetry according to the specific tumor, which type of microsphere to use for which tumor, and the best combinations with other molecular-targeted therapies.

## Figures and Tables

**Table 1:** Patient characteristics and treatments before TARE.

	All	TARE as Bridging to LT			TARE as Palliative Treatment	*p* Value **
		All Bridging	LT Performed	LT not Performed		
No. of patients	167	50	14	36	117	
Gender						
male female	146 (87.4%) 21 (12.6%)	45 (90.0%) 5 (10.0%)	14 (100.0%) 0	31 (86.1%) 5 (13.9%)	101 (86.3%) 16 (13.7%)	0.616
Age (years)						
mean ± SD median, range	67.5 ± 8.4 66.6, 45.7–85.5	62.6 ± 4.5 62.6, 50.6–70.7	63.4 ± 2.5 63.0, 59.4–69.0	62.3 ± 5.0 62.6, 50.6–70.7	69.6 ± 8.7 72.0, 45.7–85.5	<0.001
HCC diagnosis to TARE (months)						
mean ± SD median, range	12.9 ± 22.4 5.3, 1.3–177.5	10.8 ± 12.7 5.5, 1.3–54.3	9.9 ± 10.1 5.7, 1.3–40.3	11.1 ± 13.4 5.5, 1.5–54.3	13.8 ± 25.4 4.9, 1.3–177.5	0.509
HCC treatments before TARE *						
none surgery transarterial chemoembolization percutaneous radiation systemic therapy liver transplantation	83 (49.7%) 33 (19.8%) 59 (35.3%) 4 (2.4%) 12 (7.2%) 2 (1.2%)	23 (46.0%) 12 (24.0%) 17 (34.0%) 0 3 (6.0%) 0	6 (42.9%) 2 (14.3%) 6 (42.9%) 0 1 (7.1%) 0	17 (47.2%) 10 (27.8%) 11 (30.6%) 0 2 (5.6%) 0	60 (51.3%) 21 (17.9%) 42 (35.9%) 4 (3.4%) 9 (7.7%) 2 (1.7%)	0.613 0.399 0.861 0.318 1.000 1.000
HCC stage						
IA IB II IIIA IIIB IVA	1 (0.6%) 5 (3.0%) 94 (56.3%) 47 (28.1%) 13 (7.8%) 7 (4.2%)	0 0 30 (60.0%) 13 (26.0%) 6 (12.0%) 1 (2.0%)	0 0 8 (57.1%) 3 (21.4%) 3 (21.4%) 0	0 0 22 (61.1%) 10 (27.8%) 3 (8.3%) 1 (2.8%)	1 (0.9%) 5 (4.3%) 64 (54.7%) 34 (29.1%) 7 (6.0%) 6 (5.1%)	0.647
Child–Pugh stage/score						
A5 A6 B7 B8	47 (28.1%) 104 (62.3%) 14 (8.4%) 2 (1.2%)	18 (36.0%) 29 (58.0%) 2 (4.0%) 1 (2.0%)	5 (35.7%) 9 (64.3) 0 0	13 (36.1%) 20 (55.6%) 2 (5.6%) 1 (2.8%)	29 (24.8%) 75 (64.1%) 12 (10.3%) 1 (0.9%)	0.253

HCC, hepatocellular carcinoma; LT, liver transplantation; TARE, transarterial radioembolization; SD, standard deviation. * multiple treatments per patient are possible; ** comparison of bridging patients (column 3) with palliative patients (column 6).

**Table 2:** Characteristics of TARE procedures.

	All	TARE as Bridging to LT			TARE as Palliative Treatment	*p* Value **
		All Bridging	LT Performed	LT not Performed		
No. of TARE procedures	245	71	16	55	174	
Treated liver lobes						
right left	170 (69.4%) 75 (30.6%)	51 (71.8%) 20 (28.2%)	11 (68.8%) 5 (31.2%)	40 (72.7%) 15 (27.3%)	119 (68.4%) 55 (31.6%)	0.649
Treatment cycles						
all monolobar bilobar sequential	209 173 (82.8%) 36 (17.2%)	61 51 (83.6%) 10 (16.4%)	14 12 (86.7%) 2 (14.3%)	47 39 (83.0%) 8 (17.0%)	148 122 (82.4%) 26 (17.6%)	0.999
No. of procedures per patient						
1 2 3 4	101 (60.5%) 57 (34.1%) 6 (3.6%) 3 (1.8%)	30 (60.0%) 19 (38.0%) 1 (2.0%) 0	12 (85.7%) 2 (14.3%) 0 0	18 (50.0%) 17 (47.2%) 1 (2.8%) 0	71 (60.7%) 38 (32.5%) 5 (4.3%) 3 (2.6%)	0.763
Tumor burden in target volume (%)						
mean ± SD median, range	18.0 ± 22.2 7.4, 1.7–100	12.7 ± 19.1 5.6, 1.7–100.0	10.4 ± 11.4 5.3, 1.7–37.6	13.5 ± 21.4 6.2, 1.9–100.0	20.2 ± 23.2 8.8, 1.7–100.0	0.013
Microsphere type						
^90^Y glass ^90^Y resin ^166^Ho PLLA	158 (64.5%) 84 (34.3%) 3 (1.2%)	48 (66.2%) 26 (33.8%) 0	11 (68.8%) 5 (31.2%) 0	36 (65.5%) 19 (34.5%) 0	111 (63.8%) 60 (34.5%) 3 (1.7%)	0.814
Prescribed activity (GBq; median, range)						
^90^Y glass ^90^Y resin ^166^Ho PLLA	2.1, 0.4–7.7 1.0, 0.3–3.1 1.8, 1.4–5.6	2.1, 0.6–7.7 1.0, 0.6–2.0 -	1.9, 0.6–4.3 0.85, 0.6–1.4 -	2.1, 0.6–7.7 1.0, 0.6–2.0 -	2.2, 0.4–7.1 1.0, 0.3–3.1 1.8, 1.4–5.6	0.697 0.881 -

LT, liver transplantation; TARE, transarterial radioembolization; SD, standard deviation; PLLA, poly-L-lactic acid. ** comparison of bridging patients (column 3) with palliative patients (column 6).

**Table 3:** Treatments and outcome after TARE.

	All	TARE as Bridging to LT			TARE as Palliative Treatment	*p* Value **
		All Bridging	LT Performed	LT not Performed		
No. of patients	167	50	14	36	117	
Follow-up (months) after TARE						
mean ± SD median, range	21.3 ± 19.4 14.5, 0.9–112.6	26.7 ± 23.5 21.8, 1.4–100.6	45.7 ± 30.2 38.1, 5.0–100.6	19.3 ± 14.8 14.7, 1.4–70.7	19.1 ± 16.8 13.8, 0.9–112.6	0.040
HCC treatments after TARE *						
none transarterial chemoembolization percutaneous radiation systemic therapy surgery (without LT) liver transplantation (LT)	101 (60.5%) 31 (18.6%) 19 (11.4%) 34 (20.4%) 4 (2.4%) 14 (8.4%)	20 (40.0%) 12 (24.0%) 9 (18.0%) 13 (26.0%) 1 (2.0%) 14 (28.0%)	0 4 (28.6%) 3 (21.4%) 3 (21.4%) 0 14 (100%)	20 (55.6%) 8 (22.2%) 6 (16.7%) 10 (27.8%) 1 (2.8%) 0	81 (69.2%) 19 (16.2%) 10 (8.5%) 21 (17.9%) 3 (2.6%) 0	<0.001 0.279 0.108 0.294 0.999
Occurrence of extrahepatic metastases	35 (21.0%)	9 (18.0%)	3 (21.4%)	6 (16.7%)	26 (22.2%)	0.679
Progression-free survival						
progression (events, %) median (months, 95% CI) est. PFS rate after 6 months (95% CI) est. PFS rate after 12 months (95% CI)	109 (%) 11.0 (8.9–15.1) 74.0% (66.3–80.2%) 47.5% (38.9–55.5%)	34 (68.0%) 10.0 (7.2–16.3) 72.2% (56.9–82.8%) 47.4% (32.4–61.0%)	5 (35.7%) - (4.3-.) 71.4% (40.6–88.2%) 63.5% (33.1–83.0%)	29 (80.6%) 9.2 (7.1–13.7) 72.7% (54.0–84.8%) 41.1% (24.2–57.2%)	75 (64.1%) 11.4 (7.7–17.8) 74.8% (65.5–82.0%) 47.5% (37.1–57.2%)	0.033
Overall survival						
death (events, %) median (months, 95% CI) est. survival rate after 6 months (95% CI) est. survival rate after 12 months (95% CI)	125 (74.9%) 16.6 (13.2–21.8) 82.0% (75.3–87.1%) 60.5% (52.6–67.4%)	33 (66.0%) 23.1 (15.0–31.7) 86.0% (72.9–93.1%) 74.0% (59.5–84.0%)	3 (21.4%) - 92.9% (59.1–99.0%) 92.9% (59.1–99.0%)	30 (83.3%) 19.8 (11.1–23.9) 83.3% (66.6–92.1%) 66.7% (48.8–79.5%)	92 (78.6%) 13.9 (10.8–17.8) 80.3% (71.9–86.5%) 54.7% (45.3–63.2%)	0.001

HCC, hepatocellular carcinoma; LT, liver transplantation; TARE, transarterial radioembolization; SD, standard deviation; CI, confidence interval. * multiple treatments per patient are possible; ** comparison of bridging patients (column 3) with palliative patients (column 6).

## Data Availability

The data can be shared up on request.
